# Institutionalizing Innovation: From Pilot to Scale for Co-Packaged Oral Rehydration Salts and Zinc—A Case Study in Zambia

**DOI:** 10.9745/GHSP-D-23-00286

**Published:** 2024-02-28

**Authors:** Jane Berry, Simon Berry, Elizabeth Chizema, Bonface Fundafunda, Davidson H. Hamer, Stephen Tembo, Rohit Ramchandani

**Affiliations:** a ColaLife, London, United Kingdom.; b African Leaders Malaria Alliance, Zambia End Malaria Council; Formerly of Zambian Ministry of Health, Lusaka, Zambia.; c Africa Resource Centre; Formerly of Medical Stores Limited, Lusaka, Zambia.; d Department of Global Health, Boston University School of Public Health, Boston, MA, USA.; e Section of Infectious Diseases, Department of Medicine, Boston University Chobanian & Avedisian School of Medicine, Boston, MA, USA.; f Ruralnet Associates Ltd., Lusaka, Zambia.; g Department of International Health, Johns Hopkins Bloomberg School of Public Health, Baltimore, MD, USA.; h School of Public Health Sciences, University of Waterloo, Waterloo, Canada.; i Balsillie School of International Affairs, Waterloo, Canada.

## Abstract

We document the development and institutionalization in Zambia of a health innovation for diarrhea treatment aimed at children aged younger than 5 years: a unique oral rehydration salts and zinc (ORSZ) co-pack. Seven recommendations from the World Health Organization/ExpandNet are used retrospectively to analyze and describe the successful scale-up of this innovation from its concept stage, including in-country expansion and policy, institutional, and regulatory changes. The 7 recommendations comprise using a participatory process, tailoring to the country context, designing research to test the innovation, testing the innovation, identifying success factors, and scaling up. The scale-up of co-packaged ORSZ in Zambia is shown to be sustainable. Five years after donor funding ended in 2018, an independent, local manufacturer continues to supply the private and public sectors on a commercially viable basis. Furthermore, national coverage of ORSZ increased from less than 1% in 2012 to 34% in 2018. A key success factor was the continuous facilitation over 8 years (spanning planning, trial, evaluation, and scale-up) by a learning and steering group chaired by the Ministry of Health, open to all and focused on learning transfer and ongoing alignment with other initiatives. Other success factors included a long lead-in of inclusive initial consultation, ideation, and planning with all key stakeholders to build on and mobilize existing resources, knowledge, structures, and systems; alignment with government policy; thorough testing and radical review of the product and its value chain before scale-up, including manufacture, distribution, policy, and regulatory matters; and adoption by the government of a co-packaging strategy to ensure cases of childhood diarrhea are treated with ORSZ. With appropriate local adaptations, this approach to scale-up could be replicated in other low- and middle-income countries as a strategy to increase coverage of ORSZ and potentially other health products.

## Abstract (precis)

A multisector partnership developed a locally contextualized and owned holistic approach to project design and implementation; this process provided a strong learning platform to take a novel yet simple lifesaving health product from trial to sustainable scale-up.

## Teaser

**Key Findings:**

- We describe a highly participatory approach to improve the design and distribution of a novel oral rehydration salts and zinc (ORSZ) co-pack to treat diarrhea in children aged younger than 5 years in Zambia.
- Developing existing structures and experience across multiple sectors was key to the evolving intervention design, shared vision, resulting partnership, and sustainability.
- Aiming from the outset to embed a successful product for private sector manufacture, we suppressed funder and project branding, testing the product and its value chain.
- The process led to the successful horizontal scale-up (replication/expansion) and vertical scale-up (institutionalization) of the ORSZ co-pack.

**Key Implications:**

- By involving a broad, local stakeholder group, including both public and private partners, from planning through to scale-up, health care innovations can be successfully scaled up in a locally owned and sustainable manner.
- This approach to scale-up, with appropriate local contextualization, could be replicated in other low- and middle-income countries to increase the coverage of novel lifesaving therapies.

## INTRODUCTION

Diarrhea remains the second-leading cause of infectious-disease-related childhood mortality globally, responsible for more than 480,000 deaths annually of children aged younger than 5 years,^1^ despite the effectiveness of the well-known, simple co-therapy of oral rehydration salts and zinc (ORSZ). Since the World Health Organization (WHO) and UNICEF first called for ORSZ to be used for the treatment of childhood diarrhea in 2004,^2^ there has been insufficient scale-up of ORSZ at a global level. Global coverage currently remains at around 15%^3^; it is estimated that at current rates of scale-up, it will take more than 30 years to reach 80% coverage.^3^

Although the case study we describe here focuses on institutionalizing innovation in childhood diarrhea therapy, it offers wider lessons for scale-up. It has been framed in response to calls for examples of successful transitions to scale of health innovations that have achieved population health impact.^4^ WHO, the U.S. Agency for International Development (USAID), and others have noted the lack of well-documented case studies to support learning and analysis.^5^ Successful scale-up and sustained progress of public health interventions following an initial pilot phase is uncommon. Gupta et al.,^6^ reviewing 69 articles, found that fewer than 5% of drug or technology innovations reach scale and are sustained, while successful efforts take, on average, 14 years and cost US$2 billion. Over the past 15 years, definitions of what constitutes scale-up and models and pathways to analyze and achieve sustainable impact have been developing.^5^^,^^7^^,^^8^ USAID’s Center for Accelerating Innovation and Impact (CII) takes a business-minded approach to 5 possible scale-up models.^9^ Broadly corresponding to CII model 2 (multistakeholder partnerships), WHO advocates for a participatory process involving multiple actors, interest groups, and organizations while planning from the outset for mainstreaming.^5^ The ExpandNet initiative,^10^ whose goal is to enhance the practice and science of scale-up,^5^^,^^11^^–^^14^ captures and codifies successful examples of scale-up and has called for more case studies that follow or retrospectively describe its frameworks.^5^^,^^11^^–^^14^ While we only became aware of ExpandNet after the projects were complete, we find our approach matched their recommendations of how to increase the scalability of an innovation.^5^ Thus, although we did not follow the ExpandNet framework per se, we use the 7 recommendations as a structure here, in the following descriptive headings: use a participatory process, tailor the innovation to the context, design research to test the innovation, test the innovation, identify success features to streamline and replicate, plan and embed key changes for user organizations, and initiate scaling up.

The case study we describe focuses on institutionalizing innovation in childhood diarrhea therapy but offers wider lessons for scale-up.

## METHODS

From 2010, ColaLife,^15^ a small United Kingdom-based innovation charity, deployed a catalytic approach to improve the design^16^ and distribution^17^ of ORSZ in a kit or co-pack for the treatment of diarrhea in children aged younger than 5 years, working with end users, stakeholders, implementation partners, and product designers, both in Zambia and at the global level.

Here, we retrospectively document the progress made in Zambia using the WHO/ExpandNet recommendations. These closely align with our implementation approach, from the initial concept of the co-pack to its adoption in 2018 by the Government of the Republic of Zambia and transfer to private-sector ownership. The implementation partnership, under the Zambian Ministry of Health (MOH), included UNICEF Zambia, Keepers Zambia Foundation (KZF), and local private-sector partners with expertise in pharmaceutical manufacturing, the distribution of fast-moving consumer goods, and emerging digital communications. Implementation was supported and managed by a resource team^5^ provided by the Common Market for Eastern and Southern Africa, ColaLife, and its international supporters.

Private-sector insights and experience were key in helping the evolving partnership to enrich potential public health innovation concepts.^17^ Some initial learning came from discussing product design and value-chain thinking with The Coca-Cola Company. ColaLife’s name was inspired by the realization that Coca-Cola beverages were widely available but lifesaving medicines were not. Although this input from the Coca-Cola Company provoked some criticism,^18^ insights were provided pro bono; furthermore, the company had no commercial interest and did not fund the trial, scale-up, or the work of ColaLife.

ColaLife’s ethos, approach, and processes were originally drawn from its founders’ experience in the reorientation of an Integrated Rural Development Project^19^ in Zambia in the 1980s, influenced by the Brandt Report^20^ and by their subsequent experience of participatory community development and stakeholder engagement in the United Kingdom, synthesized into what Nilsen^21^ referred to as a “common sense approach” to innovation. ColaLife works strictly in a catalytic and enabling role with no direct implementation capacity.

This case study summarizes the quasi-experimental trial of the novel ORSZ co-pack and distribution innovations (reported elsewhere)^16^^,^^17^^,^^22^^–^^24^ and highlights the success factors in the modus operandi and implementation style to describe how scale-up was achieved between 2010 and 2018. Aligning with ExpandNet’s definitions of horizontal and vertical scale-up,^5^ we define horizontal scale-up as replication or expansion of innovations and vertical scale-up as institutionalization (i.e., changing policy, legal, and/or regulatory frameworks). Employed in tandem, these dimensions can result in sustainable scaling up.^5^ From 2018 to date, localized production, procurement, and distribution of the product in Zambia has happened independently.

Another case study (unpublished) arising from our work documents the progress toward global, vertical scale-up (an extended conceptualization of the ExpandNet model) of co-packaged ORSZ following a successful bid to amend the WHO Model Essential Medicines List^25^ and the subsequent founding in 2021 of the ORS/Zinc Co-pack Alliance,^26^ whose aim is to accelerate the uptake of the new ORSZ co-packaging recommendation globally.

## FINDINGS

### Use a Participatory Process

Between 2010 and 2011, ColaLife leadership visited Zambia 3 times, acting as innovators/catalysts with 3 main aims to: review the operating environment in-country, meet decision-makers and stakeholders to gauge interest, and meet end users. Gupta et al.^6^ suggest that alignment of these 3 contextual elements is critical to the success of any innovation. Three highly interactive in-country workshops explored responses to a novel ORSZ co-pack and new distribution models to improve access. The areas explored included alignment with current initiatives and past learning, product design, and value-chain development, with a new concept to deliver co-packs from the pharmaceutical manufacturer to rural community retailers serving end users, exploiting the unused space between the necks of Coca-Cola bottles transported in crates.^17^ The workshops were open to and attended by representatives of multiple potential user organizations, including the MOH, local and international nongovernmental organizations (NGOs), and the private sector. Project implementation partners self-selected from this process, resulting in a co-written funding plan and a memorandum of understanding with the MOH. Funding was secured via the United Kingdom’s Department for International Development (DFID, now the Foreign, Commonwealth & Development Office) and match-funded by an award made to ColaLife after a competitive Innovation Bootcamp hosted by Johnson & Johnson/Janssen in 2011.^27^

### Tailor the Innovation to the Context

A design-thinking process^16^ synthesized wide-ranging stakeholder input, including from end users, to inform the utility and feasibility of product design, local manufacture, and distribution. Before selecting Zambia, this process had evolved during a period of 2 years (2008 to 2010). Possible trial countries were considered based on the following criteria: a high burden of diarrhea in children aged younger than 5 years; ease of doing business; political stability and safe operations; government’s favorable attitude toward public-private partnerships for health; English speaking; and reasonable mobile phone coverage. Zambia met these criteria. With a poorly developed retail pharmacy network (just 59 retail pharmacies countrywide in 2011, with all but 19 of them in the capital, Lusaka),^28^ the MOH was developing a policy for accredited drugstores^29^ and, thus, supported a trial using rural community retailers. Given appropriate training and development, these retailers’ potential as access points for simple, over-the-counter medicines in underserved areas has been noted by others.^30^ The MOH also favored exploring a public-private delivery role for the parastatal medicines distributor, Medical Stores Ltd. (MSL), now known as Zambia Medical Supplies Agency.

The design-thinking process continued for a further 9 months in-country to explore complementarity and alignment with, for example, community health worker (CHW) training programs,^31^ local manufacturing expertise, experience with the use of discount vouchers,^32^ and the use of mobile phones and tablets for retailer support and project monitoring. The final implementation partnership (UNICEF, KZF, Pharmanova, SABMiller, Zoona, and MSL) was serviced by ColaLife staff seconded to Common Market for Eastern and Southern Africa, the accountable body for the funding contribution of DFID’s Zambia Office. A participatory learning and steering group (LSG) was established and met quarterly under the chairmanship of the MOH to offer guidance and continuing linkages to initiatives across Zambia and provide a forum to synthesize and transfer learnings (including challenges and failures). Attendance was open to implementation partners and any organization interested in the vision, whatever their sector or thematic area, and more than 20 organizations participated. Meetings rotated among multiple host organizations who wished to share their own work, including John Snow Inc., Clinton Health Access Initiative, the Centre for Infectious Disease Research in Zambia, and USAID.

Before launching distribution of the novel ORSZ co-pack, we interviewed retailers and wholesalers and conducted focus groups with end users to refine the final product design, branding, and pricing.^16^ We tested information, education, and communication materials based on prior good practice examples in the region; these were duly approved by the MOH for use with consumers, retailers, and CHWs.

We interviewed retailers and wholesalers and conducted focus groups with end users to refine the final product design, branding, and pricing.

The final product design, branded “Kit Yamoyo” (which translates as “Kit of Life” in several local languages), was approved by the Pharmaceutical Regulatory Authority (now the Zambia Medicines Regulatory Authority) for trial marketing and distribution. It contained an imported, boxed blister-pack of 10 dispersible pediatric zinc tablets (as none were made in-country at that time) and 8 novel, locally manufactured, orange-flavored ORS sachets (4.2 g per sachet), for which the co-pack container measured the required 200 mL of water to make up the solution correctly. This novel ORS sachet size was identified by public health stakeholders we had consulted and in earlier literature^33^ as being more appropriate than the traditional 1 L sachets for the home treatment of diarrhea as they better matched 1 child’s daily consumption. The trial co-pack also contained handwashing soap. The low-osmolarity ORS powder was formulated and packed, and the co-packs were assembled locally by our manufacturing partner, Pharmanova (Zambia) Ltd., to sell into the market on a trial basis.

Pricing along the value chain was set based on a target retail price of 5 Zambian kwacha (ZMK) (just under US$1 at that time), noted as an acceptable price in caregiver focus groups and for expected retailer and wholesaler margins for similar products. An innovative cost/price mechanism reversed the usual “cost plus” pricing model, whereby acceptable margins are added to the cost of production, instead calculating price points using a “price minus” approach that subtracted expected margins from a target retail price affordable to caregivers.^17^ This produced a target ex-factory price that, for the trial period only, was achieved by the project by providing a small subsidy to the manufacturer, with a view of eliminating this subsidy post-trial through economies of scale and cost savings. The benefit of subsidizing the ex-factory price was that the value chain and price points used in the trial were the same as those envisaged for the scale-up. This meant that the trial tested both the product and its value chain.

### Design Research to Test the Innovation

The ColaLife Operational Trial in Zambia (COTZ) aimed to test the hypothesis that uptake of ORSZ combination therapy for childhood diarrhea can be significantly increased in rural communities by simultaneously creating an innovative co-packaged ORSZ product together with its value chain.^17^ Value-chain thinking in private-sector distribution for public health remains an emerging field. The research, considered a core part of the innovation process from the outset and supported by an executive role within ColaLife, tested the impact of creating an aspirational, easy-to-use ORSZ co-pack, generating demand through marketing, and making it profitable for all those involved in fulfilling that demand: manufacturer, wholesalers, and community-level rural retailers. The co-created COTZ plan to test both the impact of the product design and the co-distribution synthesized local stakeholder workshop outputs with those from the Johnson & Johnson Innovation Bootcamp,^27^ as well as best practice from UNICEF Zambia (particularly evaluation), DFID, and the technical packaging partners, PI Global Limited and Amcor. Additionally, a wider, pro bono resource team included crowdsourced voluntary expertise in product design and distribution. The rigorously designed quasi-experimental study received the requisite ethical clearances, with further details, including primary and secondary outcomes, sample size calculations, and statistical analyses, presented elsewhere.^16^^,^^17^

### Test the Innovation

The COTZ distribution of Kit Yamoyo ran for 12 months, from August 2012 to August 2013. The Principal Investigator, in collaboration with UNICEF Zambia, led the evaluation, managing an external research team.^23^ Baseline, midline, and endline surveys were conducted, and the 2 rural intervention districts, Kalomo and Katete, were each matched with a comparator district (Monze and Petauke, respectively). Intervention districts were selected according to various criteria, including diarrhea burden, rurality, mobile phone coverage (to support the extension-based retailer support system), and the presence of an independent, general wholesaler interested in participating.^17^

A commercially designed value chain operated from the outset. The manufacturing partner produced the trial co-pack, which could be ordered by participating wholesalers and delivered under a commercial arrangement with MSL, operating their usual public health delivery schedules to the intervention districts. It was then purchased by existing small retailers at the community level, who were trained by project staff and had been signposted to the wholesale supply. Retailers sold in communities where marketing and sensitization took place throughout the COTZ via CHW activities, radio talks and jingles, dramas, and posters. Key messages were developed, tested, and refined during the trial, and subsequently, and included, “Be a wise mother” and “Be wise, be prepared, beat diarrhoea.” In each of the 2 intervention districts, training was conducted using open-source ColaLife-produced resources^34^ (aligned with other initiatives’ ongoing training activities) to introduce the project and its aims and cover product-specific and general benefits of ORS and zinc and related health issues. The training was tailored for various audiences: local councils and traditional leaders, 60 existing CHWs (30 in each implementation district), 2 wholesalers (1 in each district), and 96 village retailers (50 in Kalomo and 46 in Katete). CHWs also distributed vouchers during the first 6 months to “kick-start” the value chain by ensuring from the outset that there was demand at the end of the value chain. Of the 23,982 vouchers distributed, 10,415 were redeemed. Over the 12 months of COTZ distribution, retailers bought 26,735 kits to sell in their communities, giving a ratio of cash sales to voucher sales of 61:39.^17^ The trial’s emphasis was on learning to enable a private sector business case for profit-driven scale-up to be developed in the future. Willingness to pay remained a key consideration.^35^^,^^36^

### Identify Key Success Features to Streamline and Replicate

Findings from the COTZ^17^^,^^22^^–^^24^ validated the value-chain approach and many of the product innovations. In 2014, a review of the product design was undertaken (Table 1) in preparation for scale-up to incorporate learning into a viable commercialization and reduce the ex-factory price of the co-pack to eliminate the trial subsidy. The partnership proposed target pricing and design efficiencies but agreed that the manufacturer remained free to determine its own costing and pricing in commercial confidence. This decision allowed the manufacturer to prepare to manage commercially sustainable scale-up in the future. We mitigated against risk of noncontinuation by making all designs and learning open source so other manufacturers (in Zambia or elsewhere) could benefit.

**TABLE 1.** How Learnings From the ColaLife Operational Trial in Zambia Influenced Product Design for Scale-Up

**Innovation/Aspect**	**Trial Learning/Evidence**	**Action**
Co-packaging ORS and zinc	Both elements were delivered together, increasing coverage of WHO-recommended co-therapy.^17^	Co-packaging of ORS and zinc retained.
Correct mixing of ORS	93% of Kit Yamoyo users reported mixing the ORS correctly, using the 200 mL sachets provided and the packaging as a measuring vessel, compared with 60% of users who used a traditional 1 L ORS sachet.^16^	Measuring functionality retained.
Number of ORS sachets in a co-pack	75% of endline respondents who used Kit Yamoyo said they only used 4 (or fewer) of the 8 200 mL ORS sachets provided. A further 10% used all 8 sachets.^16^	Number of 200 mL sachets in co-pack reduced from 8 to 4 to achieve cost savings and match usage data.
Duration of ORS dosing	Kit Yamoyo users who used 4 sachets at endline reported similar length of ORS use compared with users of 2 1 L sachets.^16^	200 mL ORS sachet size maintained.
Perception of ORS as an effective treatment for diarrhea	Among Kit Yamoyo users this increased by 14%, from 78% to 92%.^23^	ORS flavoring and color retained; co-packaging of ORS and zinc retained.
Packaging design for co-distribution	During the trial, only between 4% and 8% of retailers^24^ chose to nest co-packs between bottles in Coca-Cola crates while distributing over the “last mile,” from wholesaler to retail outlet (a trip typically undertaken on a bicycle, on foot, or on the back of a truck).	Trial packaging designed to fit in Coca-Cola crates was dropped in favor of a cheaper, gusseted bag that could be used to measure the volume of water required to mix the ORS correctly; it is also more amenable to being manufactured locally.
Zinc use	Among children with diarrhea in the intervention districts, zinc use increased from less than 1% pretrial to 46% at endline.^17^	Co-packaging of ORS and zinc retained.
Zinc adherence	Findings suggested the need for better messaging related to zinc adherence. While endline respondents could cite correct usage (“A complete 10-day course should be administered” was cited by 55% in Kalomo and 27% in Katete), just 46% and 25% of Kit Yamoyo users in Kalomo and Katete, respectively, reported actually adhering to the full 10-day regimen.^23^	The blister pack for the zinc tablets was redesigned, with the aim of increasing adherence. This was facilitated by the fact that the local manufacturer began manufacturing zinc tablets immediately after the trial.
Multilingual information, education, and communication leaflet/instructions	Just 30% of Kit Yamoyo users at endline reported using the instructions.^24^ It was noted that reading skills, where they exist, are usually in English rather than in indigenous languages, of which there are more than 70 in Zambia.	For scale-up, a simplified, graphics-based patient information leaflet in English only was used.
Handwashing soap	Post-trial, Zambia’s Revenue Authority ruled that a co-pack containing soap would attract value-added tax on the entire product.	Removed the soap bar from the low-cost flexi-pack, targeted at the lower end of the market, to avoid value-added tax. The soap was retained in the more expensive, screw-top format.

Abbreviations: ORS, oral rehydration salts; WHO, World Health Organization.

#### Product Design and Benefits

A high level of product satisfaction was reported both among retailers interviewed and by respondents in the endline survey.^23^ Among Kit Yamoyo users, all said they would use ORS again the next time their child had diarrhea (n=173) and 99% (95% confidence interval (CI): 97.2, 100) noted they would use Kit Yamoyo specifically.^24^ Among retailers interviewed, 92% in Kalomo and 97% in Katete said they planned to continue selling Kit Yamoyo.^23^ Key aspects of the product design that were demonstrated to benefit users were thus retained in a comprehensive redesign of the co-pack. The key findings and actions taken for scale-up are summarized in Table 1.

All Kit Yamoyo users surveyed said they would use ORS again the next time their child had diarrhea, and 99% of them noted they would use Kit Yamoyo specifically.

**FIGURE 1:**
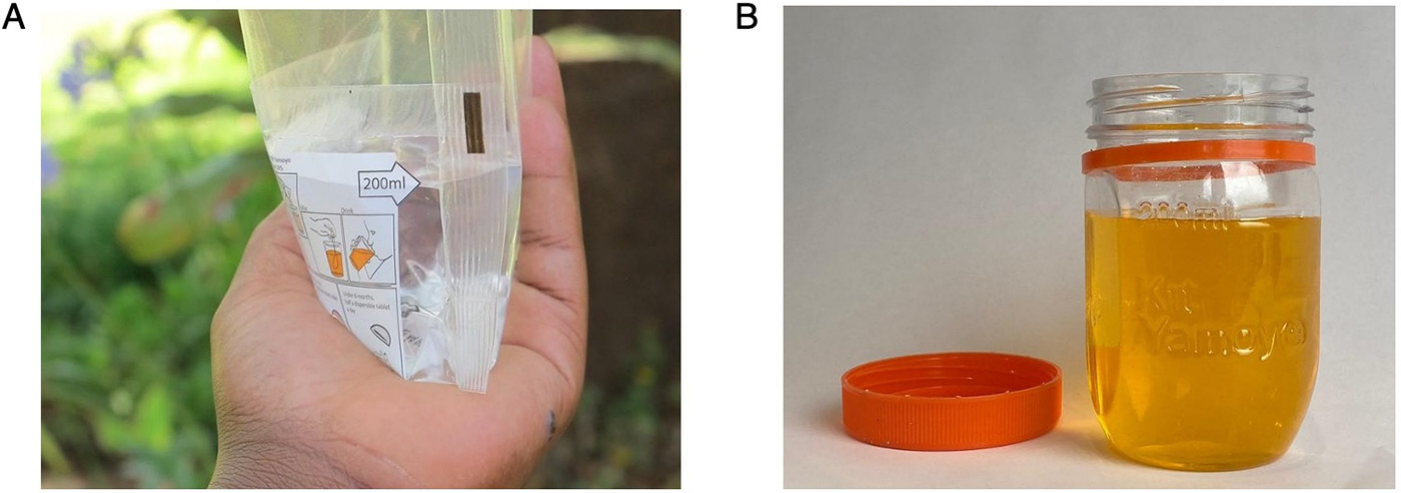
Both Oral Rehydration Salts and Zinc Co-Pack Kit Yamoyo Scale-Up Formats in Zambia Retained the Measuring Functionality (A) Plastic Flexi-Pack and (B) Plastic Screw-Top Jar^16^

As indicated in Table 1, a design aspect of the intervention that was not taken into the scale-up phase was the bespoke, imported packaging that allowed “nesting” in Coca-Cola crates to facilitate transport. It was replaced by a cheaper, locally manufactured plastic screw-top jar and a simple plastic “flexi-pack.” Each of these scale-up co-pack formats was designed to measure the required 200 mL of water to correctly dissolve the contents of each ORS sachet (Figure 1).

**FIGURE 2:**
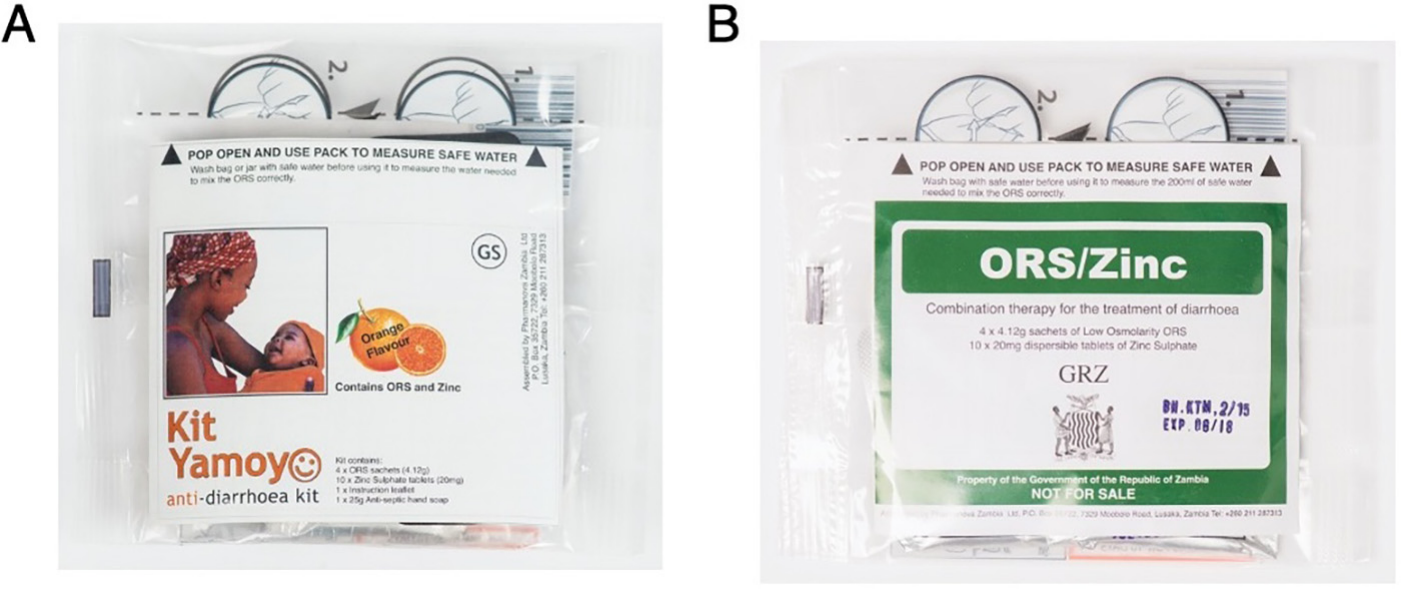
The Scale-Up Oral Rehydration Salts and Zinc Co-Packs Use the Same Flexi-Pack Packaging (A) Kit Yamoyo and (B) Government of the Republic of Zambia Branded Version

In a post-project open tender, the Government of the Republic of Zambia selected the flexi-pack format for its own branded product for free distribution via public-sector clinics that provide free health care. The transparent packaging design was retained and meant that the branding could be changed by swapping the information, education, and communication leaflets without the expense of printing new packaging (Figure 2).

#### Costing and Pricing

From the outset, the aim of long-term sustainability, coupled with a value-chain approach, underpinned all aspects of planning. This offered novel areas of learning. The value-chain pricing assumptions tested were validated. Vouchers, although useful to kick-start the value chain, were complex to administer in the longer term. They were deployed for 6 months for commercial value chain establishment in the trial and scale-up, where they comprised approximately one-third of retail sales in each case (Table 2).

**TABLE 2.** How Learning From the ColaLife Operational Trial in Zambia Influenced Costing and Pricing for Scale-Up

**Innovation/Aspect**	**Trial Learning/Evidence**	**Action**
“Price minus” costing: value-chain price points for the product and ex-factory target price derived from willingness to pay and expected margins.	Margins and RRP were acceptable to retailers, wholesalers, and end users.^17^	Trial pricing and margins retained for scale-up; ex-factory subsidy successfully removed (see below).
Restrict use of subsidy to the reduction of the ex-factory price to allow expected margins and achieve a retail price that matched willingness to pay.	Design modifications based on trial findings (Table 1) reduced the cost of production.	Manufacturer deployed savings to reduce the ex-factory price and eliminate the subsidy, to avoid having to change the price points, value chain, and market expectations established during the trial.
Through the learning and steering group, involve the MOH in understanding the process of developing a private-sector product and its benefits.	Product benefits fully understood by the MOH.	Toward the end of the scale-up phase, the MOH commissioned a government-branded version of the co-pack for distribution, free of charge, through health centers.
Use of discount vouchers during the first 6 months of COTZ distribution, to prime the value chain.	“Scratch card” vouchers redeemable by mobile phone were complicated to administer. Cessation of vouchers 6 months into distribution did not unduly undermine demand at the COTZ trial RRP.^6^	Electronically managed discount vouchers were costly/unsustainable and ruled out of scale-up plans, although simple paper vouchers were deployed to kick-start the value chain in scale-up projects.
Cost of social marketing/marketing.	It was too ambitious to fully establish the brand within a 12-month trial, even in the trial areas.	Funded scale-up projects continued social marketing, with the manufacturer gradually taking on some commercial marketing by the end of scale-up and fully from 2018 onward, although their investment capacity in marketing is low.
Cost of retailer training/CHW training in product benefits/basic health issues.	Product benefits and basic health issues were well retained, both by trained retailers and CHWs. COTZ training materials (large-scale booklets for use at public meetings) were too costly for scale-up.	Scale-up training materials reduced to 10 illustrated flash cards^39^ (Figure 5); these were offered as open-source materials. Knowledge of the product’s benefits began to be embedded with health staff/CHWs by the end of scale-up and now form part of CHWs’ practice.^40^

Abbreviations: CHW, community health worker; COTZ, ColaLife Operational Trial in Zambia; MOH, Ministry of Health; ORS, oral rehydration salts; RRP, recommended retail price.

### Plan and Embed Key Changes for User Organizations

Two early aspirations achieved were an agreement on the transfer of intellectual property to local commercial ownership and a change of strategy by the MOH to use co-packaged ORSZ to increase the use of both ORS and zinc for the home treatment of diarrhea in children aged younger than 5 years.

Despite the COTZ’s success in Zambia, initial attempts to secure a single source of funding for a nationwide scale-up were unsuccessful. Despite this hiatus, the partnership between ColaLife and local stakeholders remained strong; local stakeholders continued planning for scale-up, with ColaLife acting in a support role and coordinating a pro bono resource team. Funding for subnational scale-up was eventually secured and combined with a successful but unfunded effort to get Kit Yamoyo on the shelves of the nationwide supermarket chain in Zambia (see Initiate Scaling).

To support the scale-up planning effort from their United Kingdom base, ColaLife undertook several activities, with quarterly coordination visits to Zambia. The quarterly LSG chaired by the MOH continued to share various aspects of the endline analysis and offer a platform for others to share their relevant experience and learning.

ColaLife also continued capacity-building with the local NGO partner (KZF), including staff training, monitoring and evaluation planning and reporting, development and use of a key performance indicator dashboard^37^ and other tools, support with reports to funders, and data analysis. Liaising with key stakeholders in the MOH, ColaLife emphasized Zambian ownership of the innovation and advocated that co-packaged ORSZ be included on the Zambian Essential Medicines List (EML) and procurement listings to facilitate vertical scale-up.

Additionally, ColaLife supported the manufacturer to redesign the co-pack packaging and contents based on the COTZ findings and to obtain final approvals of the post-trial co-pack formats with the Zambia Medicines Regulatory Authority (Table 1). Data analysis was conducted by ColaLife to support the decision to reduce the number of ORS sachets in the co-pack from 8 to 4, another key factor in cost reduction.

**FIGURE 3:**
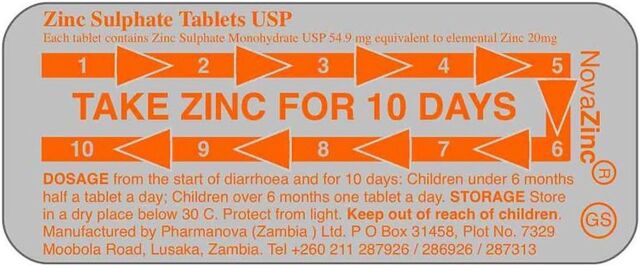
Design Improvements Adopted by Manufacturer for Zinc Tablet Blister Pack to Improve Adherence to the 10-Day Regime

ColaLife deployed its own funding to source design support to produce ideas for a blister pack for a locally manufactured zinc tablet (Figure 3), with clearer usage instructions, a simplified information leaflet, and packaging artwork. The effectiveness of this redesign has not yet been tested. All production decisions and responsibility for costing and pricing were taken by the manufacturer. These efforts aimed to support the scale-up of the COTZ innovation after the end of the trial to increase access to lifesaving treatment for children with diarrhea in Zambia.

### Initiate Scaling

Scale-up in Zambia included horizontal (expansion/replication) and vertical (regulatory, policy, political, legal, and institutional) scale-up.

**FIGURE 4:**
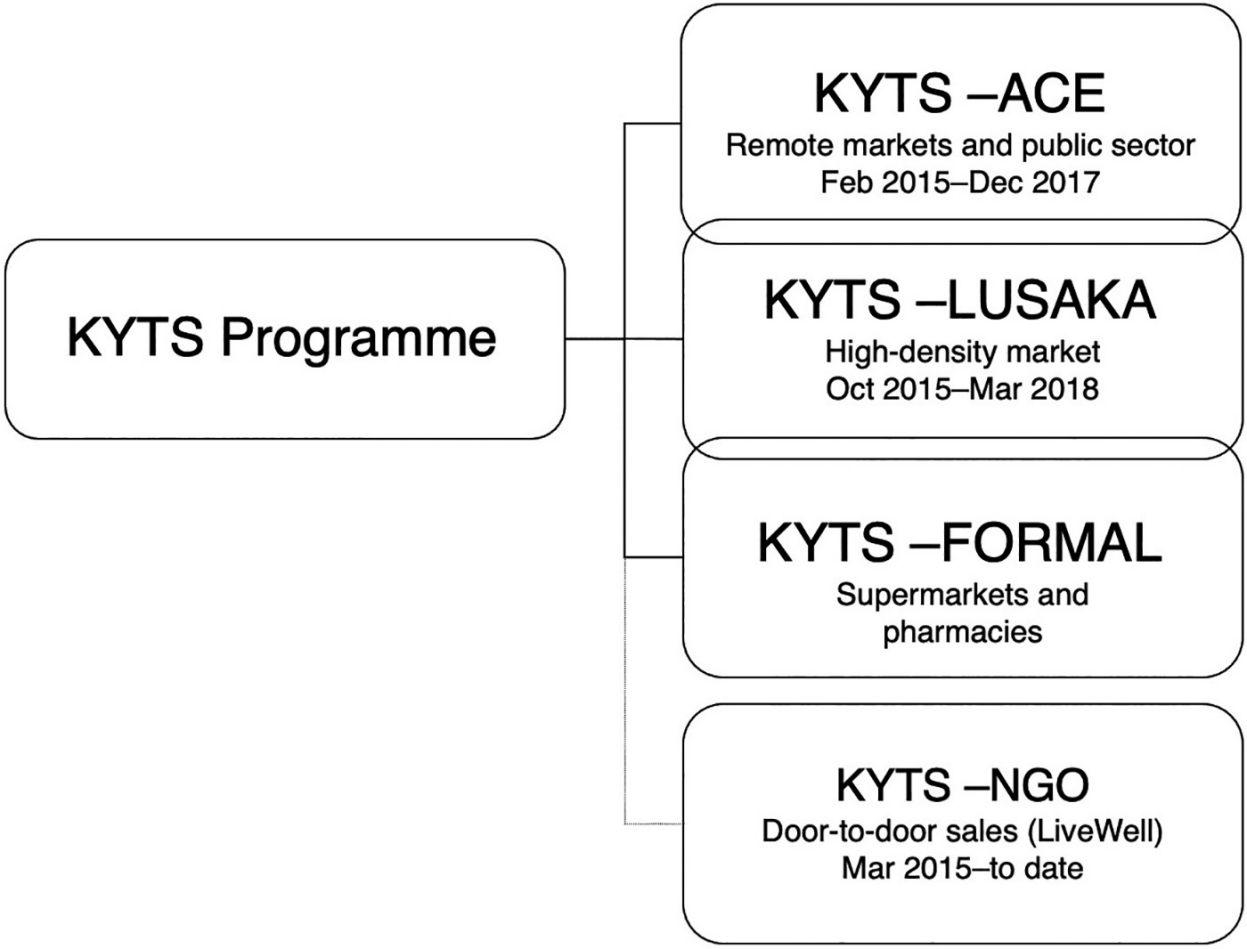
The Structure of the Kit Yamoyo Transition to Scale Programme Abbreviations: KYTS, Kit Yamoyo Transition to Scale; NGO, nongovernmental organization.

Following the COTZ, ColaLife’s coordination and learning facilitation role continued under MOH oversight through the ongoing LSG. As bids for nationwide funding failed (2014–2015), the partnership, judging that further delay risked losing the momentum gained by the success of the COTZ, was forced to progress in a piecemeal fashion. The scale-up effort, called the Kit Yamoyo Transition to Scale Programme (KYTS), consisted of 4 threads: KYTS-ACE, funded by the SUN Programme and focusing on improving nutrition in hard-to-reach areas; KYTS-LUSAKA, funded by UK Aid, continued to explore value chains; and 2 unfunded/semi-commercial threads, focusing on sales to supermarkets, pharmacies, and the LiveWell NGO (Figure 4).

KYTS-ACE and KYTS-LUSAKA were subnational projects and allowed activity in 2 very different geographical foci. Under KYTS-ACE, KZF scaled up distribution of the new public-sector co-pack and trained health center personnel in a nutrition-focused project in 14 qualifying remote/rural districts.^38^ This effort included 452,000 government-branded co-packs (Figure 2) funded by the scale-up project being distributed through MSL’s usual procedures. The training was supplemented by limited retailer training in 10 of these districts to promote the private-sector product.

In Lusaka Province, the private-sector development project, KYTS-LUSAKA, was led by ColaLife and implemented by KZF. This project focused on value-chain development and retailer support and training and was underpinned by product familiarization for health center staff but with no distribution of the new public-sector format.

The manufacturing partner led 2 unfunded initiatives: (1) KYTS-FORMAL, distribution via the Shoprite supermarket chain and pharmacies, and (2) KYTS-NGO, selling of Kit Yamoyo to a new social enterprise (LiveWell) offering door-to-door sales in Eastern Province. In all scale-up initiatives, kits destined for private-sector channels were purchased by wholesalers and retailers from the manufacturer with no subsidy. These market channels have continued to be commercially managed by the manufacturer since 2018 to date. The overlapping interventions are shown in Figure 4. A notable gap in the horizontal scale-up was the populous, industrialized Copperbelt Province, for which no funding was available.

**FIGURE 5:**
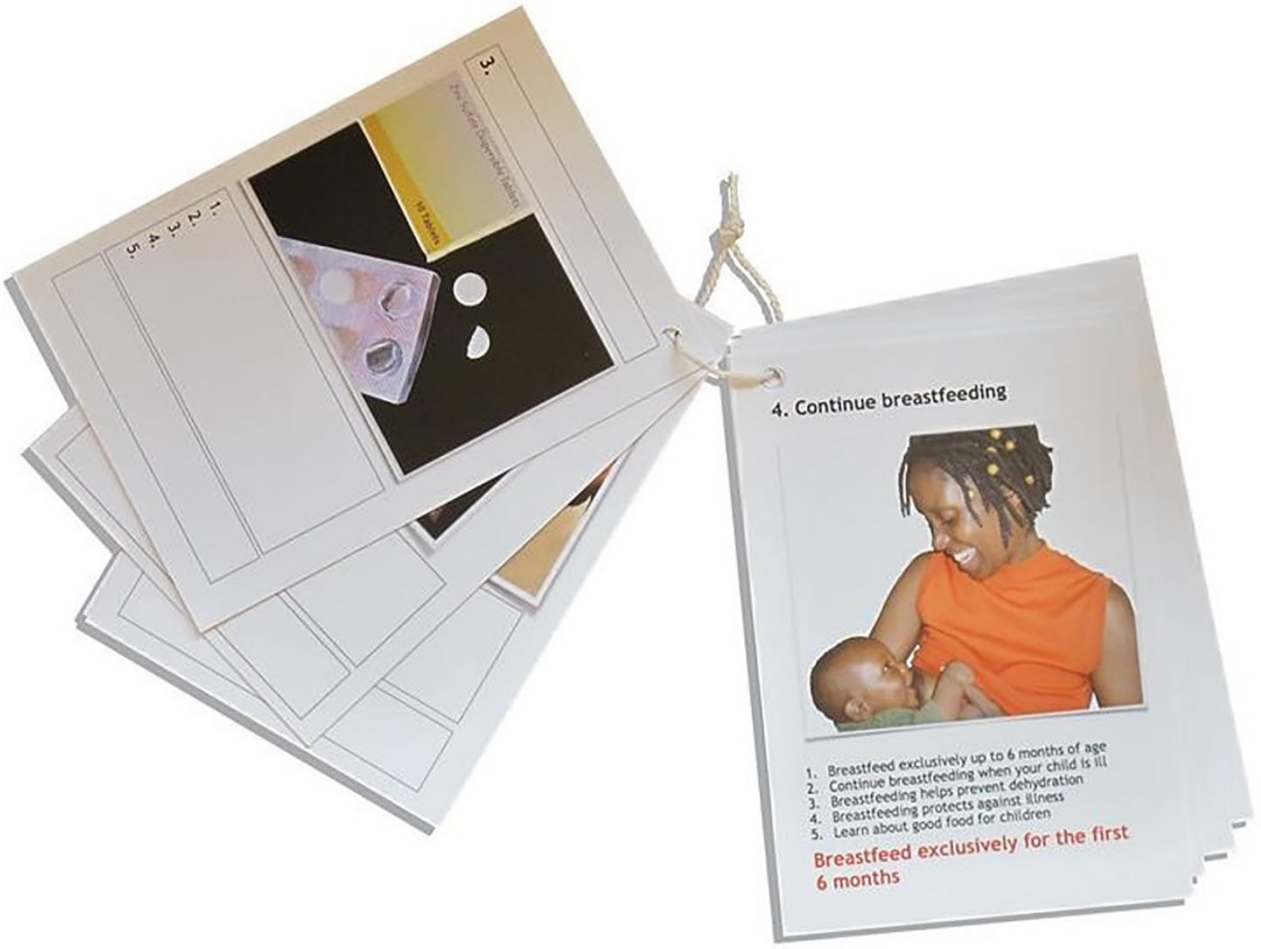
Illustrated Flash Cards Used for Training During the Scale-Up Phase of Kit Yamoyo Oral Rehydration Salts and Zinc Co-Packs in Zambia

To promote integration and clarity, all elements of the scale-up used training materials and approaches developed during the COTZ (Figure 5) and shared management information systems, dashboards, and survey tools.^39^

#### Marketing

Due to the previously mentioned limitations, a strategically planned, national scale-up marketing campaign has never been realized. While the trial and scale-up project budgets allowed some investment in marketing activities, this was largely limited to targeted project areas.

During the COTZ trial, approximately 21% of the total trial budget was dedicated to marketing, supported by government-funded CHWs providing face-to-face outreach marketing (“sensitization”) as part of their work in the community and at health-focused events in the intervention districts. Community-level retailers were supplied with posters and trained to convey product benefits to customers, with follow-up monthly support visits. These were backed with radio advertisements and features for the duration of the trial.

The KYTS-LUSAKA scale-up project, prioritizing private-sector distribution, dedicated 26% of its budget to marketing, including billboards in the city, retailer training with point-of-sale materials and wall paintings, radio jingles and features, as well as discount vouchers to incentivize the value chain reach.

The KYTS-ACE project, prioritizing free public-sector distribution, was extensively supported by sensitization through CHWs and health center staff in the 14 remote districts. Only 5% of the budget was allocated to marketing. Nevertheless, selected small retailers were trained and supported in 10 of the 14 remote/rural districts, underpinned by radio and community events. There were 5 town-based billboards for the commercial format.

Since project funding ceased in 2018, the manufacturer has had little capacity to absorb even limited ongoing marketing activities. However, it has taken advantage of occasional TV and trade fair opportunities, some community activities in the capital such as Child Health Week, and Facebook. However, promoting ORSZ for childhood diarrhea is now standard practice for CHWs in Zambia.^40^

Despite market development limitations, by the end of these scale-up projects in 2018, the national coverage of ORSZ had increased to 34%, according to the Demographic and Health Survey.^41^ This contrasts considerably with the coverage of less than 1% measured by the COTZ baseline survey in 2012.^17^ This national coverage is not all directly attributable to the intervention described here, which only intervened in 25% of Zambia’s districts. In early 2018, as the projects ended and government co-packs purchased by the KYTS-ACE project were exhausted, funding was secured for 265,000 additional co-packs as a stopgap to enable the government to organize its own procurement, which happened later that year. These were distributed nationwide according to government priorities. It should be noted that throughout the trial and scale-up, ORS and zinc were also available separately, albeit in smaller quantities from existing health center kits.

**FIGURE 6:**
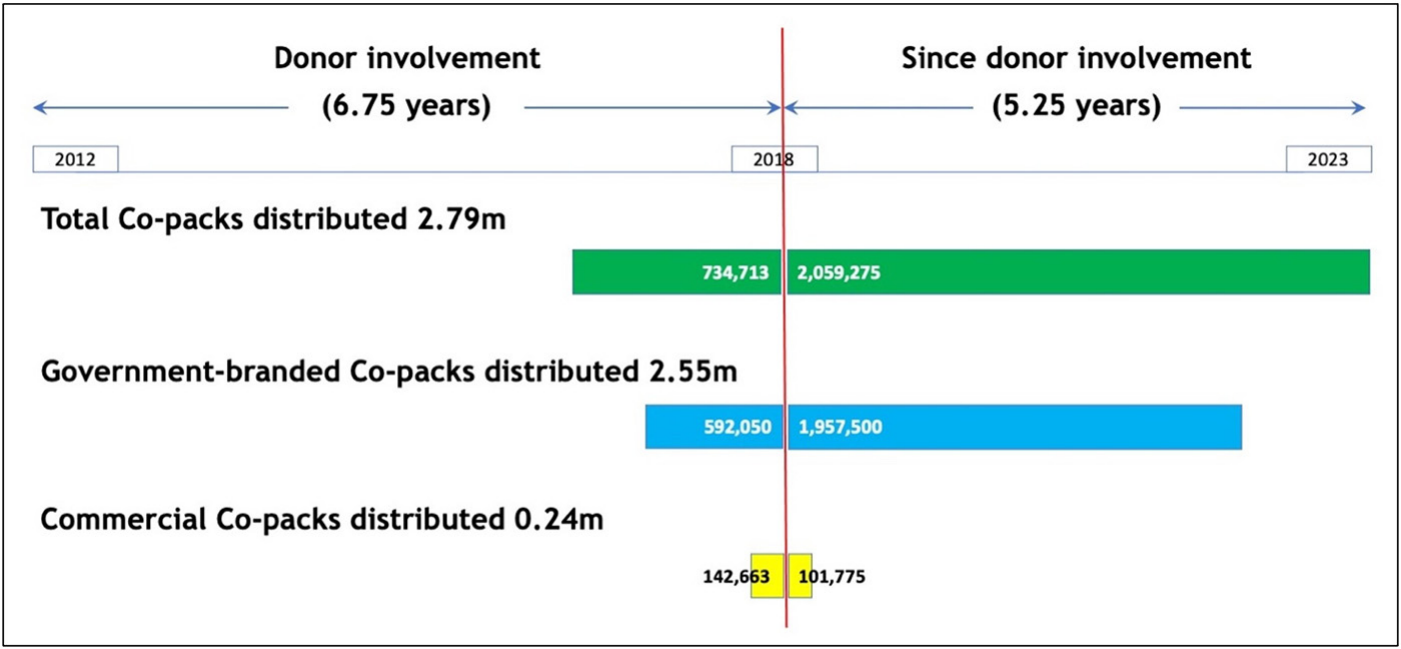
Oral Rehydration Salts and Zinc Co-Pack Sales in Zambia During Donor Involvement and Since Source: Manufacturer’s sales data.

The COTZ intervention (including planning) and scale-up project budgets together amounted to just over US$3 million, a relatively modest cost for an innovation trial and scale-up over 8 years. Since scale-up funding ceased in 2018, to date, more than 2 million kits have been sold into the market on a commercial basis with the large majority (95%) bought and distributed by the government (Figure 6).

## DISCUSSION

The process we describe led to the successful and sustainable horizontal and vertical scale-up of co-packaged ORSZ in Zambia. Although coverage in Zambia is not yet ubiquitous, 34% places Zambia with the third highest coverage in Africa.^41^ However, the move from the COTZ to scale-up was considerably underfunded and piecemeal due to funding constraints; a planned nationwide scale-up, implemented on a strategic scale, was not possible. We achieved 2 largely concurrent interventions, together covering approximately 25% of Zambia’s districts, between February 2015 and September 2018. Despite our efforts in retail channel development, the level of scale achieved has largely been due to the government adopting the co-pack and distributing it nationwide. Nonetheless, since 2018, the manufacturer has continued commercially viable production and sales of the ORSZ product in Zambia, including through some retail channels and sales to the Government of the Republic of Zambia, despite changes in government leadership and senior MOH personnel. The manufacturer’s sales data, which they have continued to share with the partnership, show that of the 2.79 million co-packs sold, 74% (2.06 million) were sold since the donor-supported scale-up programs had ended.

The process led to the successful and sustainable horizontal and vertical scale-up of co-packaged ORSZ in Zambia.

The experience in Zambia up to 2018 also provided a springboard for international vertical scale-up through a successful request to amend the WHO EML (2019 edition)^25^ and the foundation of the ORS/Zinc Co-pack Alliance.^26^

WHO noted in 2007^5^ that scaling up is both an art and a science: it involves the heart as well as the mind; is seldom a linear process of research leading to advice and then action; and is a social, political, and institutional process that engages multiple actors, interest groups, and organizations. This case study illustrates the importance of both art and science approaches, including winning hearts and minds.

WHO and ExpandNet recommend that innovation pilot projects should involve participatory design and empower user organizations. The initial consultations for the initiative described here were lengthy, broad-based, and inclusive, with a design thinking approach, contributing ultimately to successful scale-up and institutionalization in Zambia. From the beginning, both the COTZ and the subsequent scale-up projects were designed for institutionalization within the country. To achieve this, the project team aligned with existing and emerging initiatives, infrastructure, and systems, cultivating local leadership by engaging interested NGOs, the private sector, government at national and district levels, and traditional leaders. This approach emphasized the importance of future local ownership in all aspects of the initiative.

Seeking routes that lead to policy change (vertical scale-up) can embed progress. Therefore, we sought alignments with concurrent and emerging government policies, such as extending CHW training and deployment, championing of local manufacturers, enhancing the parastatal MSL, developing health shops/drugstores, and improving government rural health posts. The process ultimately triggered a key policy change: a pledge from the MOH to revise the national EML as a route to institutionalization. However, national processes for institutionalization can be unclear, slow, and unpredictable. Although EMLs can offer a useful route to help embed treatment innovations in policy, revisions can take several years. The Zambia experience illustrates that strategic change can happen without formal EML revision, provided policy change is embedded in institutions. This change was made possible in Zambia due to the commitment of the then Minister of Health and the Permanent Secretary, stemming from their early and continuing involvement, particularly their leadership of the LSG.

In this case study, the LSG was a crucial factor for the trial’s success and its transition to scale. It was established and facilitated by ColaLife, emerging from the trial design workshops in 2010–2011 and running until the end of scale-up interventions in 2018. The group included a wide range of actors beyond the implementation partners. ColaLife provided its own funding to ensure the LSG continued to meet regularly throughout the funding hiatus post-trial and during the scale-up phases. The inclusive membership and shared learning function embedded in the group helped to sustain participation from civil society groups, the private sector, large international NGOs, and small local NGOs, ensuring long-term ownership and responsibility. To encourage local ownership, ColaLife’s branding and, wherever possible, funder branding were omitted from customer-facing products and materials. A resource team provided by ColaLife supported advocacy and technical progress, particularly in the analysis of learning and open data-sharing. Focusing partners on their shared, ongoing vision rather than on a single lead organization or funding opportunity helped to keep the partnership and learning group together, promoting sustainability.

The institutionalization pathway benefited from robust data obtained from the COTZ^17^ and the scale-up projects, which enabled real-time “course adjustments” when necessary. These data included both quantitative and qualitative inputs (e.g., household surveys and interviews with health professionals, retailers, and users). We found that rigorous analysis of lessons learned was needed, but the strong relationships established helped prepare partners for radical overhauls of design, distribution, training, and other elements while highlighting key success factors. One such success was the value chain that was subsequently effectively exploited by our private-sector partner.^17^ Subsidizing at the ex-factory price point during the COTZ was a key decision. It meant that the trial could establish and test the value chain envisaged for the subsequent scale-up while meeting the purely commercial pricing and profit expectations of actors further along the chain.

Even with successful innovation trials, it can be difficult to source funding for effective scale-up (in-country or internationally). Despite our scale-up funding challenges, we demonstrate that an inclusive process combined with a drive to enable and support viable, locally owned commercialization (unlike many international NGO development models) is key. Empowering the manufacturer to take ownership of the intellectual property and make production and costing decisions at an early stage enabled them to play a key role, commercially, in supporting the national scale-up effort. The private-sector market for ORSZ, developed during the COTZ and subsequent scale-up interventions, was small and has remained so, compared with large orders procured by the government. However, on the supply side, an ongoing private market has encouraged the manufacturer to maintain a continuous production line, putting it in a very strong position to win large government orders when these arise periodically. On the demand side, access to simple medicines has many aspects that benefit from a whole-market approach, including sensitization, social marketing, and retailer training, costs that a small manufacturer cannot cover. However, as this case study shows, stimulating a private-sector value chain can provide a small but important complementary supply of customers, as well as rich learning opportunities.^17^^,^^22^

## CONCLUSION

The ColaLife experience in Zambia has shown that a novel approach to health care innovation can be successfully scaled up and, crucially, that by adopting such approaches, we can increase the coverage of a simple, lifesaving therapy in a locally owned and sustainable manner. This model of scale-up could be replicated, with local contextualization, in other low- and middle-income countries as a strategy to increase the coverage of ORSZ and other lifesaving therapies.

## Notes

**Peer Reviewed**

**First published online: **January 19, 2024.

## References

[1] Diarrhoea. UNICEF. Updated December 2022. Accessed December 11, 2023. https://data.unicef.org/topic/child-health/diarrhoeal-disease

[2] World Health Organization (WHO). *Clinical Management of Acute Diarrhoea: WHO/UNICEF Joint Statement*. WHO; 2004. Accessed December 11, 2023. https://apps.who.int/iris/handle/10665/68627

[3] Data & evidence: cost of co-packaged ORS and zinc. ORS and Zinc Co-pack Alliance. Accessed December 11, 2023. https://orszco-pack.org/data-evidence/#cost

[4] Simmons R, Fajans P, Ghiron L, eds. *Scaling Up Health Service Delivery: From Pilot Innovations to Policies and Programmes*. World Health Organization; 2007. Accessed December 11, 2023. https://www.who.int/publications/i/item/9789241563512

[5] World Health Organization (WHO). *Practical Guidance for Scaling Up Health Service Innovations*. WHO; 2009. Accessed December 11, 2023. http://apps.who.int/iris/bitstream/handle/10665/44180/9789241598521_eng.pdf

[6] Gupta A, Thorpe C, Bhattacharyya O, Zwarenstein M. Promoting development and uptake of health innovations: the Nose to Tail Tool. F1000 Res. 2016;5:361. 10.12688/f1000research.8145.1. 27239275 PMC4863676

[7] Barker PM, Reid A, Schall MW. A framework for scaling up health interventions: lessons from large-scale improvement initiatives in Africa. Implement Sci. 2015;11(1):12. 10.1186/s13012-016-0374-x. 26821910 PMC4731989

[8] Yamey G. Scaling up global health interventions: a proposed framework for success. PLoS Med. 2011;8(6):e1001049. 10.1371/journal.pmed.1001049. 21738450 PMC3125181

[9] USAID Center for Accelerating Innovation and Impact (CII). *Pathways to Scale: A Guide for Early-Stage Global Health Innovators on Business Models and Partnership Approaches to Scale-Up*. CII; 2016. Accessed December 11, 2023. https://2012-2017.usaid.gov/sites/default/files/documents/1864/Pathways-to-Scale-Guide_20161013_online-508.pdf

[10] ExpandNet. Accessed December 11, 2023. https://expandnet.net

[11] Fajans P, Ghiron L, Kohl R, Simmons R. *20 Questions for Developing a Scaling Up Case Study*. Management Systems International/ExpandNet/World Health Organization; 2007. Accessed December 11, 2023. https://expandnet.net/PDFs/MSI-ExpandNet-IBP%20Case%20Study%2020%20case%20study%20questions.pdf

[12] World Health Organization (WHO); ExpandNet. *Nine Steps for Developing a Scaling-Up Strategy*. WHO/ExpandNet; 2010. Accessed December 11, 2023. https://apps.who.int/iris/bitstream/handle/10665/44432/9789241500319_eng.pdf

[13] World Health Organization (WHO); ExpandNet. *Beginning With the End in Mind: Planning Pilot Projects and Other Programmatic Research for Successful Scaling Up*. WHO/ExpandNet; 2011. Accessed December 11, 2023. https://www.who.int/publications/i/item/9789241502320

[14] Newest addition to the ExpandNet toolkit: the Implementation Mapping Tool. ExpandNet. Accessed December 11, 2023. https://expandnet.net/expandnet-implementation-mapping-tool

[15] ColaLife. Accessed December 11, 2023. https://www.colalife.org

[16] Ramchandani R, Berry S, Berry J, Pratt BA, Saka A, Black RE. Design thinking to improve rational use of oral rehydration salts: lessons from an innovative co-packaged diarrhoea treatment kit. BMJ Innov. 2023;9(3):132–143. 10.1136/bmjinnov-2023-001081

[17] Ramchandani R, Berry S, Berry J, Tembo S, Black RE. Emulating value-chains of fast-moving consumer goods to improve uptake of co-packaged ORS and zinc for childhood diarrhoea: evaluation of the ColaLife trial. BMJ Innov. 2022;8(3):169–182. 10.1136/bmjinnov-2021-000914

[18] Berry S, Berry J, Ramchandani R, Spencer N. Should we welcome multinational companies’ involvement in programmes to improve child health? BMJ. 2015;350:h3046. 10.1136/bmj.h3046. 26085507

[19] Integrated Rural Development Project (IRDP) Serenje-Mpika-Chinsali (SMC). *The Integrated Rural Development Programme (IRDP), Serenje, Mpika and Chinsali (SMC): The Programme and Its Affects After 5 Years*. IRDP Occasional Paper No. 13. IRDP SMC; 1986.

[20] The Brandt Report: a summary. sharing.org. January 31, 2006. Accessed December 12, 2023. https://sharing.org/information-centre/reports/brandt-report-summary

[21] Nilsen P. Making sense of implementation theories, models and frameworks. Implement Sci. 2015;10:53. 10.1186/s13012-015-0242-0. 25895742 PMC4406164

[22] Savaget P, Henderson CJ, Evans S. *Emulating Value Chains of Consumer Goods to Save Lives: A Case Study of ColaLife’s Work in Zambia*. IBM Center for The Business of Government; 2019. Accessed December 12, 2023. https://www.businessofgovernment.org/sites/default/files/Emulating%20Value%20Chains%20of%20Consumer%20Goods%20to%20Save%20Lives_0.pdf

[23] RuralNet Associates Ltd. *ColaLife Operational Trial Zambia: Improving Use, Access, Availability and Awareness of ORS and Zinc for the Treatment of Diarrhoea in the Home - Endline Survey Report*. RuralNet Associates Ltd; 2014. Accessed December 12, 2023. https://assets.publishing.service.gov.uk/government/uploads/system/uploads/attachment_data/file/347891/Evaluation-Cola-Life-Trial-Zambia.pdf

[24] Ramchandani R. *Emulating Commercial Private-Sector Value-Chains to Improve Access to ORS and Zinc in Rural Zambia: Evaluation of the ColaLife Trial*. Dissertation. Johns Hopkins University Bloomberg School of Public Health; 2016. Accessed December 12, 2023. https://jscholarship.library.jhu.edu/handle/1774.2/39229

[25] World Health Organization (WHO). *World Health Organization Model List of Essential Medicines for Children: 7th list 2019*. WHO; 2019. Accessed December 12, 2023. https://www.who.int/publications/i/item/WHOMVPEMPIAU201907

[26] ORS and Zinc Co-pack Alliance. orszco-pack.org. Accessed December 12, 2023. https://orszco-pack.org

[27] Report to the community. Janssen. Accessed December 12, 2023. https://web.archive.org/web/20211119153557/http://www.janssen-verslag-samenleving-2013.be/en/economic-sustainability/open-innovation-model.htm

[28] Ballou-Aares D, Freitas A, Kopczak LR, et al. *Private Sector Role in Health Supply Chains: Review of the Role and Potential for Private Sector Engagement in Developing Country Health Supply Chains*. Technical Partner Paper No. 13. Dalberg Global Development Advisors/MIT-Zaragoza International Logistics Program; 2008. Accessed December 12, 2023. https://r4d.org/resources/private-sector-role-health-supply-chains/

[29] Government of Zambia. The Medicines and Allied Substances Bill, 2013. Accessed December 12, 2023. https://www.parliament.gov.zm/sites/default/files/documents/bills/The%20Medicines%20and%20Allied%20Substances%20Bill%2C%202013.PDF

[30] van Niekerk L, Chater R, Naydenova E, et al. *Social Innovation in Health: Case Studies and Lessons Learned from Low- and Middle-income Countries*. World Health Organization; 2017. Accessed December 12, 2023. https://tdr.who.int/publications/i/item/2017-09-27-social-innovation-in-health-case-studies-and-lessons-learned-from-low-and-middle-income-countries

[31] Phiri SC, Prust ML, Chibawe CP, Misapa R, van den Broek JW, Wilmink N. An exploration of facilitators and challenges in the scale-up of a national, public sector community health worker cadre in Zambia: a qualitative study. Hum Resour Health. 2017;15(1):40. 28646897 10.1186/s12960-017-0214-3 PMC5483317

[32] Bazley J. *Zoona Mobile Money: Investing for Impact (A)*. Bertha Centre for Social Innovation and Entrepreneurship; 2015. Accessed December 12, 2023. https://www.sbs.ox.ac.uk/sites/default/files/2019-01/zoona-case-a.pdf

[33] Touchette PE, Elder J, Nagiel M. How much oral rehydration solution is actually administered during home-based therapy? J Trop Med Hyg. 1990;93(1):28–34. 2304127

[34] ColaLife resources. ColaLife. Accessed December 12, 2023. https://www.colalife.org/resources

[35] IDinsight. *Mapping the Kit Yamoyo Demand Curve - Rural Monze District, Zambia*. IDinsight; 2013. Accessed December 12, 2023. https://www.colalife.org/wp-content/uploads/2021/11/00-Rural-Demand-for-Kit-Yamoyo-Final-Report-12-Nov-13-IDinsight.pdf

[36] IDinsight. *Mapping the Kit Yamoyo Demand Curve - Urban Kitwe and Ndola Districts, Zambia*. IDinsight; 2014. Accessed December 12, 2023. https://www.colalife.org/wp-content/uploads/2022/05/ColaLife-Urban-Final-Report-FINAL-copy.pdf

[37] KYTS dashboards. ColaLife. Accessed December 12, 2023. https://www.colalife.org/dashboards

[38] SUN countries - Zambia. Scaling Up Nutrition. Accessed December 12, 2023. https://scalingupnutrition.org/sun-countries/zambia

[39] Berry S. Kit Yamoyo training materials – download. ColaLife blog. November 10, 2014. Accessed December 12, 2023. https://www.colalife.org/2014/11/10/kit-yamoyo-training-materials-download

[40] Mufana CM, Akinola O, Simwanza C. Community health worker sustains continuity of essential services despite COVID-19 restrictions and community fears. Clinton Health Access Initiative blog. September 1, 2021. Accessed December 12, 2023. https://www.clintonhealthaccess.org/blog/community-health-worker-sustains-continuity-of-essential-services-despite-covid-19-restrictions-and-community-fears

[41] Zambia Statistics Agency; Zambia. Ministry of Health (MOH); ICF. *Zambia Demographic and Health Survey 2018*. Zambia Statistics Agency/MOH/ICF; 2019. Accessed December 12, 2023. https://dhsprogram.com/pubs/pdf/FR361/FR361.pdf

